# NEAT1 regulates VSMC differentiation and calcification in as long noncoding RNA NEAT1 enhances phenotypic and osteogenic switching of vascular smooth muscle cells in atherosclerosis via scaffolding EZH2

**DOI:** 10.1152/ajpcell.00587.2023

**Published:** 2024-04-22

**Authors:** Chengye Yin, Zhuowang Ge, Jiali Yuan, Yuhan Chen, Yong Tang, Yin Xiang, Yachen Zhang

**Affiliations:** Department of Cardiology, Xinhua HospitalShanghai Jiao Tong University School of Medicine, Shanghai, People’s Republic of China

**Keywords:** atherosclerosis, epigenetic regulation, NEAT1, vascular calcification, VSMC phenotypic switching

## Abstract

Atherosclerosis (AS) is a significant contributor to cardio-cerebrovascular ischemia diseases, resulting in high mortality rates worldwide. During AS, vascular smooth muscle cells (VSMCs) play a crucial role in plaque formation by undergoing phenotypic and osteogenic switching. Long noncoding RNA nuclear paraspeckle assembly transcript 1 (NEAT1) has previously been identified as a nuclear regulator that promotes tumorigenesis and metastasis, but its role in regulating VSMCs in AS remains unclear. Our study aimed to investigate the biological functions and specific mechanisms of NEAT1 in regulating VSMCs in AS. We found that NEAT1 was upregulated in the aortas of AS mouse models and dedifferentiated primary VSMCs. Silencing NEAT1 in vitro attenuated the proliferation, migration, and osteogenic differentiation of VSMCs, while NEAT1 overexpression had the opposite effect. Furthermore, NEAT1 promoted VSMC osteogenic differentiation and vascular calcification in both in vivo and in vitro vascular calcification models. We also discovered that NEAT1 directly activates enhancer of zeste homolog 2 (EZH2), an epigenetic enzyme that suppresses the expression of senescence- and antimigration-related genes, by translocating it into the nucleus. CUT&Tag assay revealed that NEAT1 guides EZH2 to the promoters of senescence-related genes (P16, P21, and TIMP3), methylating local histones to reduce their transcription. Our findings suggest that NEAT1 functions in AS by modulating the epigenetic function of EZH2, which enhances the proliferation, migration, and osteogenic differentiation of VSMCs. This study provides new insights into the molecular mechanisms underlying the pathogenesis of AS and highlights the potential of NEAT1 as a therapeutic target of AS.

**NEW & NOTEWORTHY** Our study demonstrates that the upregulation of long noncoding RNA nuclear paraspeckle assembly transcript 1 (NEAT1) promotes proliferation and migration during phenotypic switching of vascular smooth muscle cells in atherosclerosis. We also provide in vivo and in vitro evidence that NEAT1 accelerates vascular calcification. Our findings identified the direct interaction between enhancer of zeste homolog 2 (EZH2) and NEAT1 during atherosclerosis. NEAT1 is necessary for EZH2 to translocate from the cytoplasm to the nucleus, where EZH2 epigenetically inhibits the expression of genes related to senescence and antimigration.

## Graphical abstract

**Figure:**
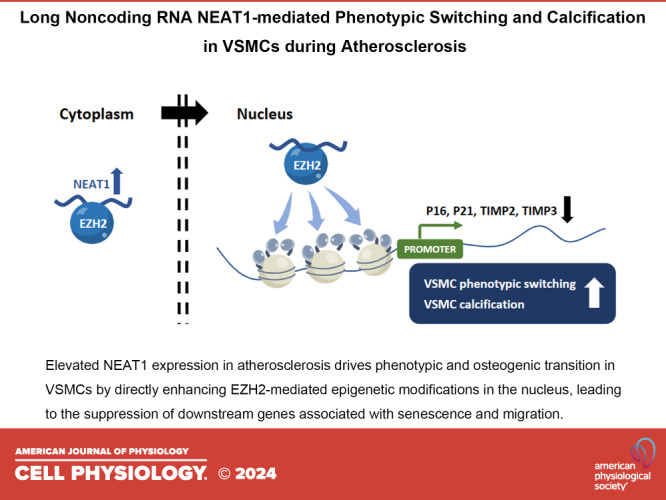

## INTRODUCTION

Atherosclerosis (AS) is a chronic inflammatory vascular disease that leads to cardio-cerebrovascular ischemia diseases (1). The major pathological processes of AS include endothelial dysfunction, immune cell infiltration, vascular smooth muscle cell (VSMC) proliferation and migration, and eventually, plaque formation and rupture (2).

VSMCs play a crucial role in the progression of atherosclerotic plaques. During AS, VSMCs can switch from a contractile to a synthetic phenotype, losing their contractile features and acquiring increased capacity in proliferation, migration, and extracellular matrix (ECM) production. This phenotypic switching is commonly considered to play an important role in promoting plaque formation in the early stages of AS (3, 4). Therefore, understanding the molecule regulation of this process during the AS progression is vital.

VSMCs also contribute to the calcification of the arterial wall, which occurs in more advanced stages of AS. Vascular calcification is the accumulation of calcium/phosphate crystals in the vessel wall, related to chronic metabolic diseases like AS, aging, and chronic renal disease (5). VSMCs that exhibit osteochondrogenic characteristics lead to arterial calcification from the early stage of AS (6). Intima calcification exacerbates AS plaque rupture, while medial calcification worsens artery stiffness. The phenotype switching of VSMCs, as mentioned above, is prevalent in vascular calcification. Synthetic VSMCs have been reported to precede the development of vascular calcification (7, 8). However, specific molecular events during this process are not fully understood.

Nuclear paraspeckle assembly transcript 1 (NEAT1) is an essential long-noncoding RNA (lncRNA) that plays a crucial role in the formation of nuclear paraspeckles (9). NEAT1 functions as an architectural scaffold by assembling protein components of nuclear paraspeckle, including non-POU domain containing octamer binding (NONO), splicing factor proline/glutamine-rich (SFPQ), and paraspeckle component 1 (PSPC1) (10, 11). In addition to its structural role, NEAT1 has also been implicated in the regulation of various diseases. Several studies have demonstrated that NEAT1 is commonly overexpressed in various types of solid tumors, such as nonsmall cell lung cancer, ovarian cancer, and hepatocellular carcinoma (12–14). The upregulation is typically associated with poor clinical outcomes such as metastasis, increased recurrence, and poor overall survival (15). NEAT1 promotes tumor progression through two mechanisms: miRNA sponge or interaction with scaffold protein such as enhancer of zeste homolog 2 (EZH2) (16). In certain cancers, NEAT1 recruits miRNAs and prevents them from inhibiting downstream gene expression, ultimately leading to enhanced tumor growth and invasion (17). Additionally, NEAT1 has been shown to recruit and directly bind to EZH2, a functional part of an epigenetic regulatory complex polycomb repressive complex 2 (PRC2), which promotes tumor cell proliferation and invasion (18, 19).

Recent research has suggested that NEAT1 may also influence cardiovascular diseases (20–22). Our team previously reported that NEAT1 could induce cardiac fibrosis in atrial fibrillation (23). In this study, we explore the potential functions of NEAT1 in VSMCs during AS. Several published studies have shown that NEAT is upregulated in AS arteries (24, 25), most of which demonstrated that NEAT1 was related to endothelial dysfunction and vascular inflammation in AS (25–28), but how NEAT1 regulates VSMC function in AS are not yet fully understood.

Our current study revealed that NEAT1 plays a crucial role in regulating VSMC functions during AS. We found that NEAT1 can affect VSMC proliferation, migration, senescence, phenotypic switching, and calcification during AS. NEAT1 alters the gene expression pattern of VSMCs by affecting the epigenetic function of EZH2. Through direct interaction, NEAT1 affects the binding of EZH2 to promoters of certain genes, thereby regulating their expression. Our study highlights the importance of NEAT1 in regulating VSMC functions and provides insight into potential targets for therapeutic intervention in AS.

## MATERIALS AND METHODS

### Animal Studies

All animal studies were approved by the Ethics Committee of Shanghai Xinhua Hospital affiliated to Shanghai Jiao Tong University School of Medicine. Specific pathogen-free C57/BL6J mice were obtained from Xinhua Hospital. All procedures were in accordance with the *Guide for the Care and Use of Laboratory Animals* (National Institutes of Health Publication, 1996).

Male adult ApoE knockout mice (8 wk old) were provided a high-fat diet (21% fat, 1.5% cholesterol) to induce atherosclerotic lesions in the aorta. After 12 wk, the mice were euthanized, and their aortas were extracted and preserved in 4% paraformaldehyde or liquid nitrogen for further use.

Male adult C57/BL6J mice (8 wk old) received a high-adenine high-phosphate diet (0.2% adenine and 1.2% phosphate) for 3 wk, followed by 10 days of calcitriol intraperitoneal administration (8.75 mg/kg/day, Sigma-Aldrich). Aorta samples were extracted and treated as mentioned above.

### Isolation and Culture of Mouse Primary Vascular Smooth Muscle Cells

Primary VSMCs (pVSMCs) were isolated from the aortas of 8-wk-old C57/BL6j mice. After euthanization, the aortas were rapidly isolated and the adventitia was dissected. The intima of the aortas was removed after longitudinal dissection. The tissue was cut into 1 mm × 1 mm pieces and was digested at 37°C for 30 min (0.744 U/mL elastase and 1 mg/mL type II collagenase). The cell suspensions were filtered by a 70-μm strainer, centrifuged at 800 *g* for 6 min, and plated on culture dishes. VSMCs were maintained in Dulbecco’s modified Eagle’s medium (HyClone, South Logan, UT) supplemented with 20% fetal bovine serum (FBS; Gibco, Grand Island, NY) and 1% penicillin/streptomycin (Beyotime, Shanghai, China) for 5 days. VSMCs were cultured and passaged in 10% FBS afterward. Cells in passages 2∼7 were used in experiments.

Calcification was induced in vitro using osteogenic medium (50 mg/L l-ascorbic acid, 10 mmol/L β-glycerophosphate, and 0.1 μmol/L dexamethasone) or control medium, both of which were administrated for 10 days. Phenotypic switching was induced using platelet-derived growth factor (PDGF)-BB (MCE, HY-P7087). VSMCs were cultured in 25 ng/mL PDGF-BB-containing medium for 48 h. GSK126 (MCE, HY-13470) was added to the medium (1 μmol/L) to inhibit the activity of EZH2.

### Antisense Oligonucleotide Transfection

Antisense oligonucleotides (ASOs) targeting Neat1 and negative control were designed and synthesized by Ribobio (Guangzhou, China). At 70% confluence, ASOs were transfected into cells using Opti-MEM medium and Lipofectamine 2000 (Invitrogen). After 24 h, cells were collected to quantify NEAT1 expression using qualitative (q)RT-PCR.

### Overexpression of Neat1 by Adenovirus Transfection

As previously mentioned (23), two Pdc315-EGFP vector-based recombinant adenoviruses were purchased from Hanbio, Co., Ltd. to overexpress Neat1 (adNeat1) and green fluorescence protein (adGFP). The stock solutions of adenoviruses were at concentrations of 1,011 plaque formation units (PFUs)/ml. The working solution of 109 PFU was applied in vitro. Transfection efficiency was measured by GFP fluorescence analysis 48 h posttransfection. In vivo, 1,011 PFU adenovirus was intravenously injected into mice (500 μL/kg) 1 wk before animal experiments. The transfection efficiency was measured by fluorescence after death.

### En Face Oil Red Staining of Whole Aorta

Lipid droplets in the tissues were visualized by staining with oil red. After euthanasia, complete aortas were isolated. Perivascular connective tissues were removed, and the arteries were dissected longitudinally. After fixation and rinse with PBS and 70% ethanol, the aortas were stained with Sudan IV staining solution for 10 min. After being rinsed with 70% ethanol 3 times, aortas were placed near a measuring scale. Images were captured using a digital camera. Statistical analysis of images was performed using Image-Pro Plus 6.0 software.

### Immunofluorescence In Situ Hybridization

Immunofluorescence in situ hybridization (IF-FISH) allows researchers to visualize protein and nuclear acid in the same sample. The isolated aortas were fixed with 4% paraformaldehyde for 24 h, embedded in paraffin, and sliced into 5-μm sections. After penetration and blocking, samples were incubated with the primary antibody ACTA2 (Abcam, ab7817), followed by fluorescently labeled secondary antibodies to enable protein visualization. Subsequently, fluorescence in situ hybridization (FISH) is performed by denaturing the samples using the FISH tag detection kit (Thermo Fisher, F32952) following previous procedures (23). The sections were incubated with fluorescently labeled oligonucleotide probes complementary to the Neat1 sequence (purchased from Servicebio, 5′- CCGCAAAGAATGATGCTCCCAGAACAAGA-3′). After being washed with hybridization washing buffer, the sections were immersed in mounting medium (Invitrogen, D1306), and the fluorescent signal produced by bound probes was visualized using a fluorescent microscope (Leica).

### CCK8 and 5-Ethynyl-2′-Deoxyuridine Staining Assay

These assays were employed to measure cell viability or proliferation. Two hundred VSMCs per well were plated in 96-well plates. CCK8 (Beyotime, C0037) was added (10 μL/well) at time points of 0, 48, and 96 h. The density of cells was reflected by the color of the media, detected by a spectrophotometer.

The 5-ethynyl-2**′**-deoxyuridine (EdU) staining was performed using the BeyoClickTM EdU Cell Proliferation Kit (Beyotime, C0071S) following the manufacturer’s guidance. Adherent VSMCs were incubated in an EdU solution, and cells undergoing division incorporated EdU with a fluorescent probe during DNA synthesis. The fluorescence was detected using a fluorescent microscope (Leica) and compared to the total cell number (DAPI) using ImageJ software.

### Transwell Migration Assay and Scratch-Wound Assay

These assays were used to measure cell migration in vitro, as described previously (23). In Transwell migration assays, 15,000 VSMCs per well were seeded in the upper chamber of a 24-well plate with a membrane containing 8-μm pores. The migration capacity was determined by the number of cells migrating towards the lower chamber. The cells passed through the membrane were fixed, stained with crystal violet, and observed under a microscope. In the scratch-wound assay, VSMCs were seeded in 6-well plates and cultured in FBS-free medium. Straight scratches were made by a sterile pipette tip. The width of the scratches was measured at 0- and 48-h time points.

### Alizarin Staining

Tissue sections or cells were fixed with 4% paraformaldehyde, dehydrated in ethanol, and treated with pH 4.2 alizarin red solution for 10 min. The samples were then rinsed with distilled water for macro- and microscopic photographing.

### Calcium Quantitation

A calcium Colorimetric Assay Kit (Beyotime, S1063S) was employed to detect the level of calcium, following the manufacturer’s instructions. Briefly, cells were collected, lysed, and added to the detection buffer. The calcium levels were assessed by colorimetry analysis, with reference to the standard curve. The protein levels were determined by using the BCA Protein Assay Kit (Beyotime, P0012), which allowed for normalization of the results to micrograms of calcium per milligrams of protein.

### Alkaline Phosphatase Activity Measurement

The Alkaline Phosphatase Assay Kit was utilized in accordance with the protocol from the manufacturers. Para-nitrophenyl phosphate (pNPP) served as a chromogenic substrate of alkaline phosphatase (ALP). The lysates of cell samples were incubated with pNPP-containing reaction buffer for 5–10 min at 37°C. The activity of ALP was measured using a spectrophotometer and normalized to the total protein concentration.

### RNA Pull-Down Assay

The Neat1 RNA pull-down assay was performed following the manufacturer’s instructions, utilizing the Pierce Magnetic RNA-Protein Pull-Down Kit (Thermo Fisher, 20164). The sense and antisense biotinylated Neat1 probes were purchased from OBiO Intelli-M (Shanghai, China). The cell lysis was incubated with the probes, and streptavidin-conjugated magnetic beads were added to the mixture to capture the probes. After being rinsed, heat denaturation, and elution, the extracted RNA-protein complexes were subjected to Western blot analysis to identify whether Ezh2 binds to Neat1.

### RIP Assay

The RIP assay was performed using the Magna RIP RNA-Binding Protein Immunoprecipitation Kit (Sigma-Aldrich, 17-700). Briefly, formaldehyde was used to crosslink the RNA-protein complex in cell samples. Cells were lysed and subjected to sonication. The lysate was then incubated with Ezh2 antibody (ABclonal, A5743) or control IgG conjugated with protein A/G magnetic beads. The RNA was subsequently isolated from the RNA-protein-antibody complex and subjected to qRT-PCR detection.

### CUT&Tag Assay

As previously described (16), the CUT&Tag assay is a novel method to detect the DNA binding to the protein. In short, cells were collected and treated with DNA Binding Library for Illumina CUT&Tag kits (Yeasen, 12598E12). The cell lysis was incubated with H3K27me3 antibody (ABclonal, A22396) or control IgG with ConA beads. The DNA segments that bind the target protein were extracted and subjected to qRT-PCR analysis using DNA spike-in as a reference. We designed primers that target promoters of mouse p16, p21, Timp2, and Timp3 with databases and tools from the National Center for Biotechnology Information.

### Western Blotting and Quantitative Real-Time PCR

Western blotting and qRT-PCR were to evaluate the expression of genes at protein and transcriptional levels, respectively. The detailed procedures were described in previous studies (23). In Western blotting, total proteins were extracted and quantified from cell or tissue samples. Proteins of various sizes were separated by SDS-PAGE and were transferred to PVDF membranes. After being blocked with nonfat milk, membranes were incubated with antibodies against Pcna (ABclonal, A0264), cyclin D1 (ABclonal, A11022), matrix metalloproteinase 2 (mmp2; ABclonal, A6247), Mmp9 (ABclonal, A2095), Opn (ABclonal, A1499), Bmp2 (ABclonal, A14708), Ezh2 (ABclonal, A5743), H3K27me3 (ABclonal, A22396), P16 (Abcam, ab108349), P21 (Abcam, ab188224), Timp2 (ABclonal, A20766), Timp3 (ABclonal, A1511), ERK1/2 (ABclonal, A4782), p-ERK1/2 (ABclonal, AP0485), Akt (abcam, ab8805), p-Akt (abcam, ab38449), Stat3 (ABclonal, A16975), and p-STAT3 (abcam, ab267373). The ImageJ software was used to analyze the grey intensity of the bands normalized to α-tubulin or Gapdh.

RNA was extracted from cell or tissue samples with TRizol (Takara, cat. no. RR036A) and reversely transcribed into cDNA using Primescript RT Master Mix kit (Takara, RR037B). SYBR dye was used to conduct the qPCR by ABI QuantStudio3 (Applied Biosystems). The ^2−ΔΔ^Ct method was applied to half-quantify the target gene mRNA expression normalized to Gapdh and 18S.

### Statistical Analysis

The data were analyzed using GraphPad Prism 9.0 and are presented as means ± SD, which was obtained from 3 independent experiments or at least six mice per group. Independent sample *t* test, one-way ANOVA with Tukey’s post hoc test, repeated-measures ANOVA with post hoc Dunnett’s or Bonferroni test, Kruskal-Wallis test followed by post hoc Dunn’s test, and Mann-Whitney *U* test were used according to different statistical conditions. *P* < 0.05 was considered as statistically significant.

## RESULTS

### Neat1 Is Upregulated in Synthetic VSMCs in Atherosclerotic Lesions

**Figure 1.**
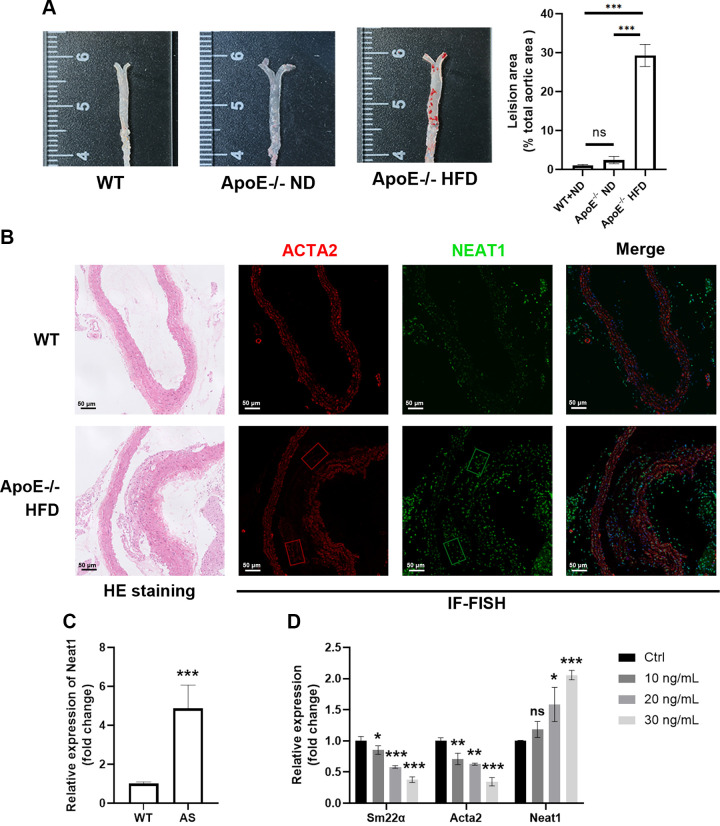
Nuclear paraspeckle assembly transcript 1 (Neat1) expression is increased in aortas of atherosclerosis (AS) mice and dedifferentiated primary vascular smooth muscle cells (pVSMCs). *A*: representative en face red oil staining of aortas from wide-type (WT) mice, ApoE knockout mice treated with normal diet (ND), and AS model induced by ApoE knockout and high-fat diet (HFD) (*n* = 6 in each group; two-way ANOVA post hoc Dunnett’s test). *B*: representative hematoxylin-eosin (HE) staining and ACTA2/Neat1 immunofluorescence in situ hybridization (IF-FISH) of AS mice aorta cross section (red, ACTA2; green, Neat1; blue, DAPI; *n* = 6 in each group). *C*: quantitative (q)RT-PCR results of mRNA expression of Neat1 in the aortas of WT and AS mice (*n* = 6 in each group) (*t* test). *D*: qRT-PCR results of mRNA expression of contractile markers (Sm22α and Acta2) and Neat1 in differentiated pVSMCs induced by PDGF-BB (*n* = 3 independent experiments; one-way ANOVA post hoc Bonferroni test and one-way ANOVA post hoc Dunnett’s test). Data are presented as means ± SE. *P < 0.05; **P < 0.01; ***P < 0.001; not significant (ns) vs. WT group or PBS.

To investigate the impact of Neat1 in AS, we established a mouse AS model induced by ApoE knockout and a Western diet with high fat and cholesterol (21% fat, 1.5% cholesterol). This chronic model dysregulates lipid metabolism, leading to artery inflammation and plaque formation. After 12 wk on the high-fat diet, significant aortic plaques were observed in AS mice after oil red staining, compared to the control group (Fig. 1*A*). We validated the presence of Neat1 expression in the aorta of AS mice via IF-FISH, an imaging technique capable of visualizing both RNA and protein within the same sample. (Fig. 1*B*) Our results demonstrate the localization of Neat1 within the nuclei of VSMCs (labeled with ACTA2), present in the tunica media and intimal plaques. In comparison to the control group, NEAT1 exhibited upregulation in the AS aortas, particularly within the shoulder region of the plaques. Furthermore, there was a noticeable accumulation of VSMCs within the same region, suggesting a potential link between increased Neat1 expression and the phenotypic switching of VSMCs during AS plaque formation. The qRT-PCR results revealed that Neat1 expression was elevated in the aorta of AS mice compared to the control group (Fig. 1*C*). To confirm this trend in vitro, we isolated the primary VSMCs from the mouse aorta and induced the dedifferentiation from a contractile phenotype to a proliferative state using PDGF-BB, which is consistent with the pathological situation of VSMCs in AS. Under this synthetic switching, the expression level of Neat1 was significantly higher (Fig. 1*D*).

### Silencing Neat1 with ASO Attenuates the Proliferation and Migration of PDGF-Treated pVSMCs

**Figure 2.**
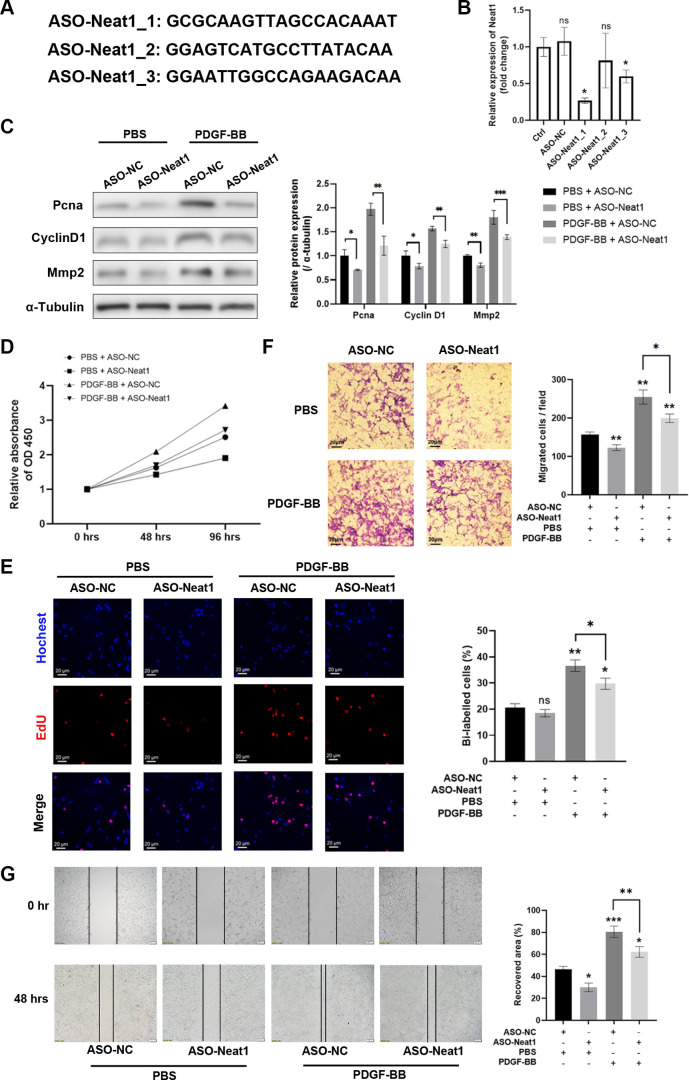
Knockdown of nuclear paraspeckle assembly transcript 1 (Neat1) attenuates the proliferation and migration of PDGF-stimulated primary vascular smooth muscle cells (pVSMCs). *A*: 3 sequences of antisense oligonucleotide (ASO) against Neat1 were constructed. *B*: quantitative RT-PCR results of Neat1 RNA level revealed that ASO-Neat1_1 had the highest knockdown efficiency among all 3. NC, negative control. *C*: protein levels of Pcan, cyclin D1, and matrix metalloproteinase 2 (Mmp2) relative to α-tubulin in ASO-transfected and PBS- and PDGF-stimulated pVSMCs (two-way ANOVA post hoc Bonferroni test). *D* and *E*: proliferation capacity of ASO-transfected and PBS- and PDGF-stimulated pVSMCs were measured by CCK8 assay and 5-ethynyl-2**′**-deoxyuridine (EdU) staining assays (Kruskal-Wallis test followed by post hoc Dunn’s test). OD, optical density. *F* and *G*: migration capacity of ASO-transfected and PBS- and PDGF-stimulated pVSMCs were measured by Transwell migration assay (Kruskal-Wallis test followed by post hoc Dunn’s test) and scratch-wound assay (two-way ANOVA post hoc Bonferroni test). Data are presented as means ± SE in 3 independent experiments. *P < 0.05; **P < 0.01; ***P < 0.001; not significant (ns) between the indicated groups.

Since Neat1 is primarily located in the nucleus, antisense oligonucleotides (ASOs) were used to downregulate Neat1 expression in vitro instead of small interfering RNA (siRNA). Three ASO sequences were designed (Fig. 2*A*), and sequence ASO-Neat1_1 was selected for subsequent experiments based on its highest knockdown efficiency (Fig. 2*B*). To investigate the role of Neat1 in VSMCs, we induced Neat1 knockdown in primary VSMCs treated with PDGF and evaluated their cellular functions. Our findings revealed that silencing Neat1 resulted in the decreased protein level of proliferation markers (Pcna and cyclin D1) and migration marker Mmp2, with or without PDGF induction, indicating a reduction in proliferation and migration (Fig. 2*C*). CCK8 assay and EdU staining results revealed that the knockdown of Neat1 significantly inhibits the proliferation of dedifferentiated VSMCs (Fig. 2, *D* and *E*). Furthermore, the silencing of Neat1 impaired PDGF-induced migration, as demonstrated by Transwell migration assay and scratch-wound assay (Fig. 2, *F* and *G*). These findings provide evidence that the deficiency of Neat1 attenuates both proliferation and migration during phenotypic switching.

### Overexpression of Neat1 Induced by Adenovirus Enhances the Proliferation and Migration of Dedifferentiated pVSMCs

**Figure 3.**
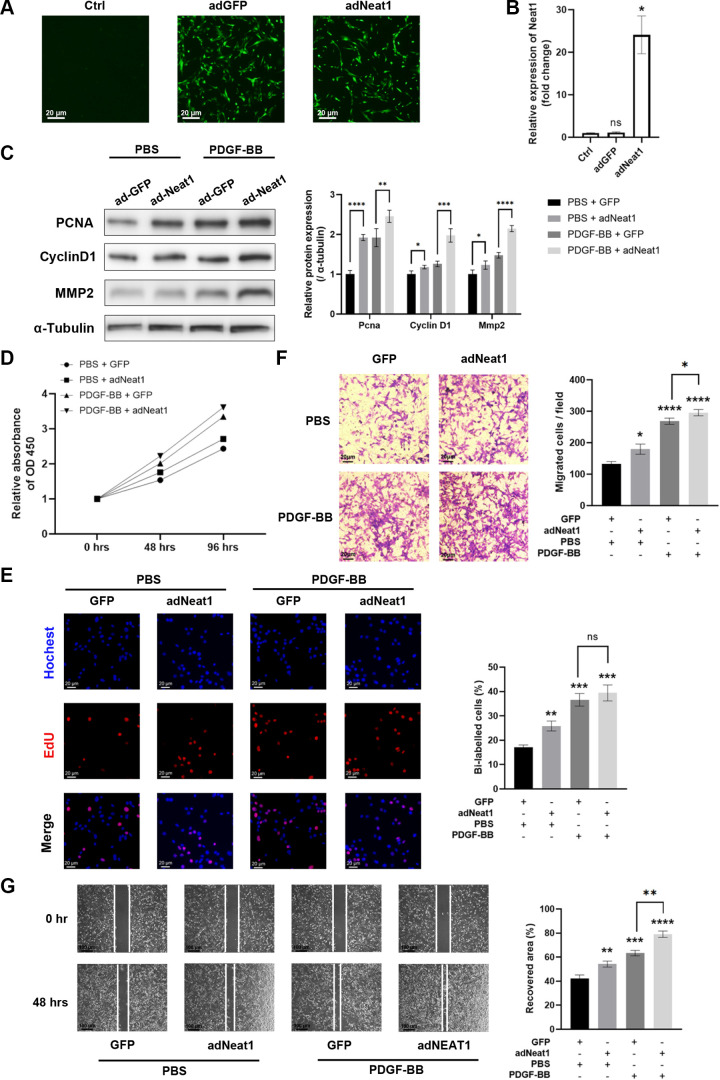
Overexpression of nuclear paraspeckle assembly transcript 1 (Neat1) promotes the proliferation and migration of PDGF-stimulated primary vascular smooth muscle cells (pVSMCs). *A*: transfection efficiency of adenovirus with the control vector green fluorescence protein (adGFP) and Neat1 overexpressing vector (adNeat1) were evaluated by green fluorescence from the GFP tag on vectors. GFP, green fluorescence protein. *B*: quantitative (q)RT-PCR results of Neat1 RNA level revealed that adNeat1 significantly overexpressed Neat1 in primary VSMCs (pVSMCs; Mann-Whitney test). *C*: protein levels of Pcan, cyclin D1, and matrix metalloproteinase 2 (Mmp2) relative to α-tubulin in antisense oligonucleotide (ASO)-transfected and PBS- and PDGF-stimulated pVSMCs (two-way ANOVA post hoc Bonferroni test, two-way ANOVA post hoc Dunnett’s test). *D* and *E*: proliferation capacity of antisense oligonucleotide (ASO)-transfected and PBS- and PDGF-stimulated pVSMCs were measured by CCK8 assay and 5-ethynyl-2**′**-deoxyuridine (EdU) staining assays (two-way ANOVA post hoc Dunnett’s test). OD, optical density. *F* and *G*: migration capacity of ASO-transfected and PBS- and PDGF-stimulated pVSMCs were measured by Transwell migration assay and scratch-wound assay (two-way ANOVA post hoc Bonferroni test). Data are presented as means ± SE in 3 independent experiments. *P < 0.05; **P < 0.01; ***P < 0.001; ****P < 0.0001; not significant (ns) between the indicated groups.

Previous studies have demonstrated the role of Neat1 in oncogenesis (19). To investigate its potential impact on injured VSMCs, we transfected a GFP-tagged Neat1 vector into pVSMCs using adenovirus (Fig. 3*A*). The overexpression fold change reached up to 20 times compared to the GFP control vector (Fig. 3*B*). Protein levels of proliferation and migration markers were further upregulated by Neat1 under PDGF treatment (Fig. 3*C*). Neat1 overexpression significantly enhanced the proliferation capacity of pVSMCs, even without dedifferentiation induced by PDGF (Fig. 3, *D* and *E*). Similarly, we observed that Neat1 overexpression promoted the migration of pVSMCs with and without PDGF treatment (Fig. 3, *F* and *G*). The above results indicate that Neat1 promotes proliferation and migration during VSMC phenotypic switching.

### Osteogenic Phenotypic Switching of VSMCs Is Regulated by Neat1 In Vitro and In Vivo

**Figure 4.**
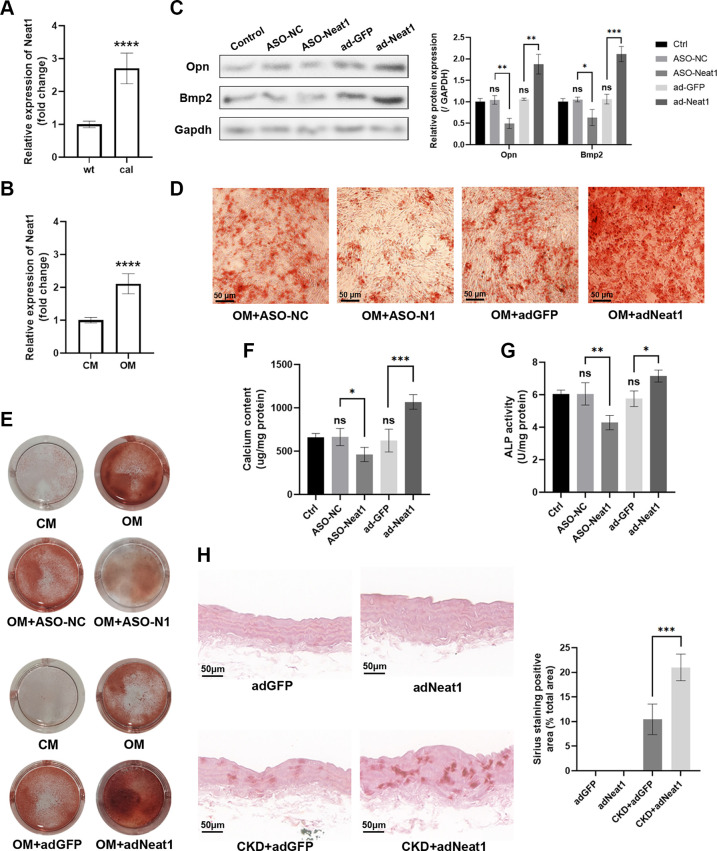
Nuclear paraspeckle assembly transcript 1 (Neat1) modulates the osteogenic differentiation of vascular smooth muscle cells (VSMCs) in vitro and in vivo. *A* and *B*: mRNA level of Neat1 in the aortas of vascular calcification model mice (cal) compared with wild-type (wt) group and primary VSMCs (pVSMCs) cultured in osteogenic medium (OM) compared with in control medium (CM) (*t* test). *C*: protein levels of osteogenic markers Opn and Bmp2 relative to Gapdh were regulated by antisense oligonucleotide-Neat1 silencing (ASO-Neat1) and overexpression (ad-Neat1) in calcified pVSMCs (one-way ANOVA post hoc Dunnett’s test). GFP, green fluorescence protein; NC, negative control. *D* and *E*: representative micro- and macroscopy images of alizarin staining of calcified pVSMCs. *F* and *G*: calcium level and alkaline phosphatase (ALP) activity of calcified pVSMCs (one-way ANOVA post hoc Dunnett’s test). *H*: representative alizarin staining results of calcified mice aortas were presented, in which Neat1 was overexpressed (adNeat1) or not (adGFP) by adenovirus (*n* = 7 in each group; Mann-Whitney test). CKD, chronic kidney disease. Data are presented as means ± SE in 3 independent experiments. *P < 0.05; **P < 0.01; ***P < 0.001; not significant (ns) between the indicated groups.

A previous study has demonstrated that Neat1 can promote osteogenic differentiation of human renal interstitial fibroblast by regulating Bmp2 expression. Calcification is a critical pathological process in the development of atherosclerotic plaques, which can enhance plaque vulnerability. Therefore, we investigated the influence of Neat1 on VSMC osteogenic differentiation. To establish a calcification mouse model, adult male mice were administered a high-adenine diet and vitamin D, which led to metabolic kidney injury and eventually aortic calcification. This form of calcification mainly occurs in the tunica media, indicating that VSMCs are primarily responsible for accumulating calcium crystals. The aortas of calcification model mice and the control group were extracted for further histological and molecular investigations. The expression of Neat1 exhibited a significant increase in the calcification aortas and cultured osteogenic pVSMCs (Fig. 4, *A* and *B*). To further investigate whether Neat1 could regulate the VSMC calcification, we down- or upregulated Neat1 in pVSMC under osteogenic conditions. Western blotting results revealed that the protein levels of Opn and Bmp2, two markers indicating the degree of calcification, showed the same trend as Neat1 expression (Fig. 4*C*). Furthermore, cultured pVSMCs were observed at both macroscopic and microscopic levels after alizarin red staining. In the osteogenic medium, the Neat1 knockdown suppressed VSMC calcification, while overexpression aggravated the calcification (Fig. 4, *D* and *E*). Neat1 knockdown reversed the calcium content and alkaline phosphatase (ALP) activity induced by osteogenic media, while Neat1 overexpression further promoted the calcium deposition (Fig. 4, *F* and *G*). To extend the in vitro findings, we upregulated Neat1 expression in vivo by adenovirus before treatment with a high-adenine diet and vitamin D. Aortic alizarin red staining indicated that Neat1 exacerbated arterial calcification and thicken the vessel wall, possibly due to its ability to promote osteogenic differentiation and proliferation (Fig. 4*H*). Overall, the results above demonstrate that Neat1 plays a crucial role in the osteogenic phenotypic switching of VSMCs.

### The Proliferation and Migration during VSMC Dedifferentiation Is Regulated by Neat1 via Ezh2

**Figure 5.**
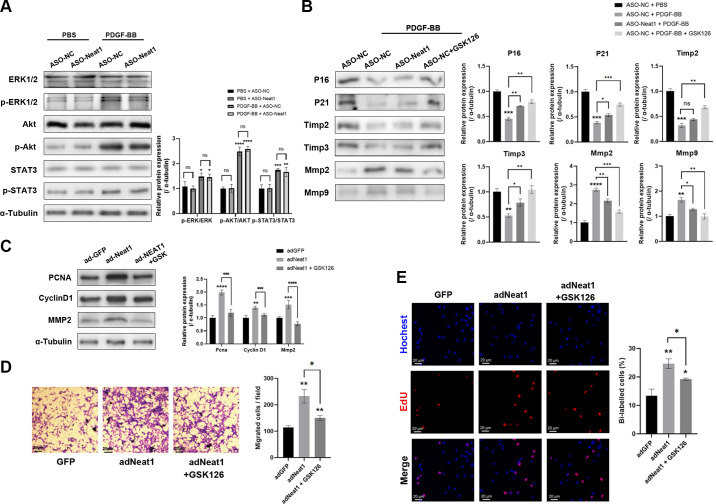
Nuclear paraspeckle assembly transcript 1 (Neat1) regulates the proliferation and migration of primary vascular smooth muscle cells (pVSMCs) via enhancer of zeste homolog 2 (Ezh2). *A*: protein levels of Erk1/2, p-Erk1/2, Akt, p-Akt, Stat3, and p-Stat3 were detected by Western blotting. pVSMCs were treated with antisense oligonucleotide (ASO)-negative control (NC) or ASO-Neat1 before treatment with PDGF-BB (two-way ANOVA post hoc Bonferroni test and two-way ANOVA post hoc Dunnett’s test). *B*: protein levels of senescence-related genes (P16, P21), antimigration-related genes [Timp2 and Timp3, inhibitors of matrix metalloproteinase 2 (Mmp2)], and migration-related genes (Mmp2 and Mmp9) were evaluated in PDGF-treated pVSMCs modulated with Neat1 knockdown and GSK126 (inhibitor of Ezh2) (two-way ANOVA post hoc Bonferroni test and two-way ANOVA post hoc Dunnett’s test). *C*: protein levels of Pcna, cyclin D1, and Mmp2 relative to α-tubulin were measured in pVSMCs treated with control adenovirus, Neat1-overexpressing adenovirus and GSK126 (inhibitor of Ezh2) together with Neat1-overexpressing adenovirus (two-way ANOVA post hoc Bonferroni test and two-way ANOVA post hoc Dunnett’s test). GFP, green fluorescence protein. *D*: Transwell migration assay revealed the migration capacity of pVSMCs modulated with Neat1 overexpression and GSK126 (two-way ANOVA post hoc Bonferroni test). *E*: 5-ethynyl-2**′**-deoxyuridine (EdU) staining demonstrated the proliferation capacity of pVSMCs modulated with Neat1 overexpression and GSK126 (two-way ANOVA post hoc Dunnett’s test). Data are presented as means ± SE in 3 independent experiments. *P < 0.05; **P < 0.01; ***P < 0.001; ****P < 0.0001; no significant (ns) between the indicated groups.

PDGF is a major ligand that promotes phenotypic switching of VSMCs in AS. It activates downstream pathways that exacerbate the proliferation and migration of VSMCs. To investigate whether NEAT1 was involved in PDGF-induced VSMC phenotypic switching, we evaluated the main downstream pathways Erk 1/2, phosphatidylinositol-3-kinase (PI3K)-Akt, and Jak/Stat during Neat1 knockdown. The phosphorylation of none of these pathways was affected by ASO-Neat1, indicating that Neat1 must promote the proliferation and migration of VSMCs through other mechanisms (Fig. 5*A*). Previous studies, including our recent work, have shown that Neat1 can directly interact with Ezh2 to regulate cardiovascular diseases (23). Ezh2 is an epigenetic enzyme that methylates Histone 3 (H3), forming the unique H3K27me3 modification at specific locations on the chromosome to suppress gene transcriptions around (16). Neat1 has been reported to scaffold with EZH2 and guide it to certain locations for specific gene suppression (18). Based on this knowledge, we hypothesized that Neat1 regulates the phenotypic switching of VSMCs via Ezh2. We assessed the protein levels of known downstream targets (P16, P21, Timp2, Timp3, Mmp2, and Mmp9) of Ezh2 and validated them using GSK126, an Ezh2 inhibitor. Western blotting results illustrated that reducing Neat1 expression enhanced the expression of genes regulated by Ezh2 (Fig. 5*B*). To further validate the role of Ezh2 in Neat1-induced phenotypic switching, we employed GSK126 to inhibit Ezh2 function. Our results demonstrated that GSK126 impaired the enhanced proliferation and migration ability induced by Neat1 overexpression, as evidenced by proliferation markers and EdU staining. Similarly, migration markers and Transwell migration assay also confirmed that Ezh2 inhibition attenuated the migration of VSMCs induced by Neat1 overexpression (Fig. 5, *C*–*E*).

### NEAT1 Directly Scaffolds EZH2 to Epigenetically Suppress Downstream Senescence- and Antimigration-Related Genes

**Figure 6.**
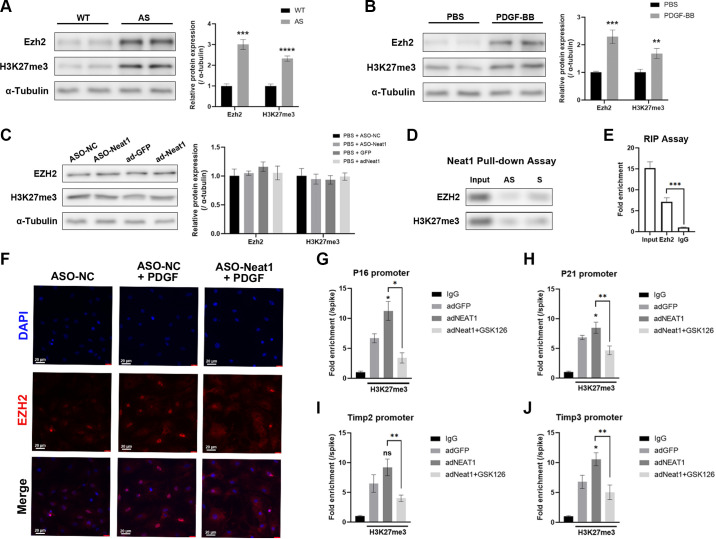
Nuclear paraspeckle assembly transcript 1 (Neat1) directly interacts with enhancer of zeste homolog 2 (Ezh2) to suppress the expression of senescence- and antimigration-related genes epigenetically. *A* and *B*: protein levels of Ezh2 and H3K27me3 were measured in the aortas of atherosclerosis (AS) mice (*n* = 5 in each group; *t* test) and PDGF-induced dedifferentiation primary vascular smooth muscle cells (pVSMCs; *n* = 3 independent experiments; one-way ANOVA with Tukey’s post hoc test). WT, wild type. *C*: protein levels of Ezh2 and H3K27me3 were examined in pVSMCs with Neat1 knocked down or overexpressed (two-way ANOVA post hoc Bonferroni test). GFP, green fluorescence protein; NC, negative control. *D*: antisense (AS) or sense (S) Neat1 probe was incubated in pVSMCs to conduct RNA pull-down assay. *E*: RIP assay proved the Ezh2 interacted with Neat1 directly (one-way ANOVA with Tukey’s post hoc test). *F*: immunofluorescence staining of EZH2 was conducted to reveal the transposition of EZH2 in pVSMC treated with PDGF-BB and Neat1-knockdown. *G*–*J*: CUT&Tag assay demonstrated the co-localization of H3K27me3 and promoters of P16, P21, Timp2, and Timp3 in pVSMCs treated with Neat1 overexpression and GSK126 (Kruskal-Wallis test followed by post hoc Dunn’s test and two-way ANOVA post hoc Dunnett’s test). Data are presented as means ± SE in 3 independent experiments. *P < 0.05; **P < 0.01; ***P < 0.001; ****P < 0.001; no significant (ns) between the indicated groups.

The precise relationship between Neat1 and Ezh2 was further investigated. We detected the expression of Ezh2 in AS aortas and PDGF-treated pVSMCs and found it was highly expressed in both situations (Fig. 6, *A* and *B*). To eliminate the possibility that Neat1 may affect Ezh2 expression, we utilized ASOs or adenovirus to manipulate the expression of Neat1. Our results demonstrated that Neat1 did not have any impact on the Ezh2 protein level (Fig. 6*C*). Nevertheless, the findings above did not exclude the possibility that Ezh2 and Neat1 might independently affect cellular functions during phenotypic switching. According to our latest work, online bioinformatic websites were used to predict that Neat1 and Ezh2 have the potential for direct interaction. Thus RNA pull-down and RIP assays were here to reveal a robust molecular interaction between Neat1 and Ezh2, suggesting a fundamental mechanism by which Neat1 regulates the proliferation and migration of VSMCs (Fig. 6, *D* and *E*).

As mentioned above, Ezh2 is an epigenetic regulator that functions in the nucleus of the cell. During phenotypic switching, more than just overexpresses in VSMCs, Ezh2 tends to translocate from cytoplasm to nucleus to conduct methylation on histones. Given the established direct interaction between NEAT1 and EZH2 without affecting EZH2 expression, we postulated that Neat1 facilitated EZH2 function in epigenetic regulation. Using the same PDGF-induced pVSMC phenotypic switching model, we observed that NEAT1 depletion prevented Ezh2 from transferring into cell nucleus, implying a crucial role of NEAT1 in guiding EZH2 to its target promoters (Fig. 6*F*). To further explore the specific binding mechanism of Ezh2 and target genes regulated by Neat1, CUT&Tag assays were performed using IgG and H3K27me3 antibodies, followed by qRT-PCR targeting specific promoters. Notably, P16, P21, Timp2, and Timp3 were selected as downstream targets of EZH2 due to their roles in cell proliferation and migration. P16 and P21 act as cell cycle inhibitors, inducing cellular senescence, while Timp3 suppresses cell migration by inhibiting various matrix metalloproteinases (MMPs). The results of CUT&Tag demonstrated elevated H3K27me modification at the promoters of P16, P21, and Timp3 upon Neat1 upregulation (Fig. 6, *C*–*F*). It was consistent with our previous observation that Neat1 knockdown reduced the protein level of P16, P21, and Timps. Since Ezh2 is the only known epigenetic enzyme responsible for epigenetic modification, these findings implied that Neat1 not only displaced Ezh2 into the nucleus but also enhanced its repressive interaction with promoters of target genes (P16, P21, and Timp3), thereby promoting proliferation and migration of cells during the phenotypic switching of VSMCs.

## DISCUSSION

In the current research, we have made a novel discovery that the long noncoding RNA Neat1 plays a critical role in the phenotypic switching of vascular smooth muscle cells (VSMCs) during the development of atherosclerosis (AS). Notably, there was a significant increase in Neat1 expression identified in the aortas of AS mice and primary VSMCs induced with platelet-derived growth factor (PDGF). According to the IF-FISH results in vivo, we found that Neat1 was present throughout the vessel wall, including the tunica media, and accumulated in the shoulder areas of plaque lesions during AS. Using antisense oligonucleotides, we successfully suppressed Neat1 expression in cultured primary VSMCs, demonstrating that Neat1 deficiency reduced the proliferation and migration capacity of VSMCs during the phenotypic switching process. Furthermore, we conducted adenovirus-mediated overexpression of Neat1 in vitro, and the results indicated that Neat1 could further enhance VSMC proliferation and migration. In addition, using aortic calcification mouse models, we demonstrated both in vivo and in vitro that Neat1 plays an integral role in the osteogenic phenotypic switching of VSMCs. Our study provided insight into the mechanism of Neat1 regulation of VSMCs and revealed that Neat1 interacts with enhancer of zeste homolog 2 (Ezh2), a vital regulator of cell proliferation and migration. Notably, Neat1 brings Ezh2 to senescence- and migration-related genes, suppressing their expression. These findings suggest a possible role for Neat1 in treating AS and other vascular diseases as a potential therapeutic target.

The role of NEAT1 in smooth muscle cells was evaluated in some previous studies across various diseases, including abdominal aortic aneurysm, pulmonary hypertension, and asthma. Similar to oncogenic studies, NEAT1 was reported to promote the proliferation and migration of smooth muscle cells. Additionally, some studies have highlighted the significance of NEAT1 in cell inflammation, oxidative stress, and senescence in SMCs (29). Our current investigation extends this line of inquiry to AS and emphasizes NEAT1’s crucial involvement in regulating VSMC functions during AS. In the progression of plaque in AS, the transformation of contractile VSMCs into a synthetic phenotype plays a crucial role (4). This transformation causes VSMCs to migrate from the tunica media to the intima and proliferate, leading to neointima hyperplasia. Meanwhile, the VSMCs aggressively produce extracellular matrix (ECM) to accelerate the formation of the plaque, while matrix metalloproteinases (MMPs) are highly released to liberate the cells. Phenotypic switching of VSMCs is a double-edged sword during AS. On the one hand, an increase in plaque size can cause severe artery occlusion, restricting the blood flow and causing recurrent ischemia symptoms like dizziness and angina. On the other hand, excessive ECM production has been considered beneficial in atherogenesis since it stabilizes the atherosclerotic plaques. Several studies have indicated that NEAT1 may play a crucial role in the progression of AS, with most focusing on endothelial cells and macrophages (24–26). Recent research has demonstrated that an overall knockout of NEAT1 attenuated the progression of AS plaque formation. However, few studies have investigated how NEAT1 regulates VSMCs. Ahmed et al. (30) reported that NEAT1 attenuates the contractile feature of VSMCs to promote phenotypic switching by scaffolding WDR5, another epigenetic enzyme. The paper suggests that increasing NEAT1 draws WDR5 away from the promoters of smooth muscle-specific contractile genes during vascular injury, eventually suppressing their expression. It also provides evidence that NEAT1, as a lncRNA, may translocate proteins responsible for posttranslational modification to regulate the cellular functions of VSMCs at an epigenetic level. Our research has revealed upregulation of NEAT1 in aortic tissues affected by AS, particularly around the shoulder area of the plaque lesions, where phenotypic switching of VSMCs predominantly occurs. Our findings demonstrate that NEAT1 plays a significant role in promoting the proliferation and migration of VSMCs, conforming to the conjecture that it may enhance the phenotypic switching of VSMCs around the surface of plaques, thereby supporting a centripetal progression of plaque formation.

Zhu et al. discovered that NEAT1 promotes the calcification of renal interstitial fibroblasts in kidney repair. This finding prompted us to investigate whether NEAT1 also regulates the osteogenic differentiation of VSMCs in AS. Through an in vitro osteogenic medium culture model and an in vivo adenine diet-induced chronic kidney disease model, we confirmed that NEAT1 promotes calcium accumulation in VSMCs, suggesting that NEAT1 upregulation in AS may contribute to arterial calcification. Calcification in AS typically occurs in the intima and is considered a risk factor for plaque rupture. However, dense calcifications like sheet-like calcification in AS lesions are more likely to stabilize the plaques rather than cause them to detach (31). Therefore, although our results show that NEAT1 can promote calcification, it is unclear whether the calcification itself is beneficial to the progression of AS. Although likely to phenotypic switching, VSMCs lose the contractile feature of smooth muscle cells, and strong in vivo and in vitro evidence supports that VSMC senescence enhances vascular calcification, while phenotypic switching is characterized by VSMC proliferation. The functions of VSMCs during AS are intricate in that both pathological processes occur during plaque formation. It is worth noticing that our results suggest that NEAT1 inhibits the senescence of VSMCs while promoting calcification. This apparent contradiction may stem from the involvement of distinct regulatory mechanisms in these two processes. Our study demonstrates that NEAT1 directly binds EZH2 and activates it to suppress the expression of senescence-related genes. Regarding the mechanism of calcification, although our study did not go further, NEAT1 was reported to promote the expression of BMP2 by sponging miRNA-129-5p. Furthermore, as mentioned earlier, NEAT1 can attenuate the contractile capacity of VSMCs, which favors osteogenic switching.

In various oncogenic studies, NEAT1 has been identified to interact with EZH2, an enzyme in the PRC2 complex responsible for the epigenetic modification in the nucleus (18). When bound by PRC2, the lysine 27 site of histone 3 was targeted, and EZH2 added three methyl groups to it to form H3K27me3. This unique histone posttranslation modification, which can only be conducted by EZH2, causes chromosome condensation around the methylation position, blocking the transcription of nearby genes. EZH2 is considered an upregulated oncogene in numerous cancers, leading to increasing H3K27me3 to repress senescence- and migration-related genes to suppress tumor growth. By activating PRC2, NEAT1 acts as an oncogene and promotes tumor development and progression in coordination with EZH2. In a recent publication, we reported that NEAT1 plays a role in regulating the fate of myofibroblasts during cardiac fibrosis (1, 23).

 Specifically, it achieves this by recruiting EZH2 to the promoter of the inhibitory Smad regulator and activating the TGF-β signaling pathway. Our current study builds on this finding by demonstrating that NEAT1 also plays a role in modulating VSMC phenotype switching. The RIP assay, RNA pull-down assay, and CUT&Tug assay together revealed that NEAT1 engages in direct interaction with EZH2. We have also demonstrated that the presence of NEAT1 is essential for the activation of EZH2, which is channeled from the cytoplasm to the nucleus by NEAT1. Once in the nucleus, EZH2 binds to the promoter regions of genes related to senescence and migration and suppresses their expression through the H3K27me3 modification. Our study reveals that NEAT1-mediated EZH2 activation targets P16, leading to the repression of VSMC senescence. In addition, EZH2 targets TIMP3, which inhibits MMPs and protects the ECM surrounding VSMCs from degradation, thereby hindering cellular migration. These results confirm and expand the previous findings regarding NEAT1’s capability to promote proliferation and migration in VSMCs.

Our current study has limitations that require further investigation. First, although we established mouse models of AS and vascular calcification, gain-of-function and loss-of-function studies of NEAT1 have not been accomplished in the AS mouse model. Conducting these experiments could provide a better understanding of the influence of NEAT1 on AS plaque formation from a histological perspective. Second, NEAT1 is a multifunctional regulator that modulates cellular functions in various ways. We only verified its function in scaffolding EZH2 to influence the epigenetics of VSMCs. Further research is necessary to explore interactions between NEAT1 and other regulators. Third, for future clinical translation, the correlation between NEAT1 and atherosclerotic lesion features must be explored. Clinical examination techniques such as cardiac CT angiography, microcomputed tomography images, and intravascular ultrasound could be used to examine the relationship between NEAT1 expression and plaque condition.

In summary, we have identified NEAT1 as an upregulated lncRNA in atherosclerosis. Through gain-of-function and loss-of-function studies, we have discovered that NEAT1 enhances the phenotypic switching and osteogenic differentiation of VSMCs. Moreover, we have found that excess NEAT1 recruits EZH2 to the promoters of P16, P21, and TIMP3, leading to the suppression of their transcription. The inhibition of senescence and enhancement of ECM degradation in VSMCs potentially accelerate the progression of atherosclerotic plaque formation. Therefore, targeting NEAT1 holds promise as a diagnostic and therapeutic strategy for AS in future clinical research.

## DATA AVAILABILITY

Data will be made available upon reasonable request.

## GRANTS

This study was supported by the National Natural Science Foundation of China Grant 82070249 (awarded to Y. Zhang) and Grant 82200453 (awarded to Y. Xiang).

## DISCLOSURES

No conflicts of interest, financial or otherwise, are declared by the authors.

## AUTHOR CONTRIBUTIONS

Z.G., Y.X., and Y.Z. conceived and designed research; C.Y. and J.Y. performed experiments; Z.G. and Y.C. analyzed data; C.Y. and J.Y. interpreted results of experiments; Z.G. and J.Y. prepared figures; C.Y. and Y.X. drafted manuscript; Y.T. and Y.Z. edited and revised manuscript; C.Y., Z.G., J.Y., Y.C., Y.T., Y.X., and Y.Z. approved final version of manuscript.
